# Supplementary material for: Are carbon emissions trading and green financial instruments synergistic? -Comprehensive quantitative research based on content analysis
Source: PLoS One. 2024 Mar 7;19(3):e0298601. doi: 10.1371/journal.pone.0298601 (PMC10919601; doi:10.1371/journal.pone.0298601)
Supplement: S1 Data — (ZIP) [file pone.0298601.s001.zip › Data/地级市Z_F工作报告.zip!/╣π╢1⁄2/├»├√╩╨/└┤╫╘╣1⁄2╓┌║┼í╛┬φ┐╦╩2╛▌═°í┐_2009.docx]

各位代表：

我代表市人民政府向大会作政府工作报告，请予审议，并请政协委员和其他列席人员提出意见。

2008年工作回顾

2008年是很不平凡的一年。在市委的正确领导下，在市人大及其常委会和市政协的监督支持下，市政府以邓小平理论和“三个代表”重要思想为指导，深入贯彻落实科学发展观，团结和依靠全市人民，继续解放思想，坚持改革开放，创新发展举措，积极克服国际金融危机的不利影响，战胜历史罕见超强台风等自然灾害，全市经济保持平稳较快发展，各项社会事业全面进步，人民生活持续改善，基本完成了市十届人大三次会议确定的目标任务。

——经济保持平稳较快增长。全年完成生产总值1217.84亿元，比上年增长9.8％（下同），其中第一产业229.5亿元，增长3.6%；第二产业506.48亿元，增长7.1%；第三产业481.86亿元，增长15%。人均GDP达到20013元，增长8.1%。来源于茂名的财政总收入161.4亿元；财政一般预算收入36.37亿元，增长14.9%。年末金融机构人民币存贷款余额分别为734亿元和220.9亿元，比年初增长18.1%和8%。全社会固定资产投资145.74亿元，增长11.5%。社会消费品零售总额499.59亿元，增长23.1%。

——现代产业体系建设步伐加快。产业结构进一步优化，非农产业的比重提高了0.52个百分点。新型工业化稳步推进，重化工业主导作用明显增强。碳五综合利用等项目建成投产，茂名石化公司2000万吨炼油扩建等30个重大项目列入省“新十项工程”，一批重化工业项目正在抓紧建设。茂名石化基地被认定为“国家火炬计划茂名石化产业基地”，博贺新港区建设正式启动，河西工业城建设积极推进。承接产业转移工作扎实有效，入园在建项目60个，投资总额13亿元。全市规模以上工业增加值282.88亿元，增长8%，其中地方工业增长14%。地方工业新增规模以上企业30家，新增产值5亿元。服务业加快发展。旅游业总收入56.59亿元。完成房地产开发投资19亿元，增长36.9%；商品房销售面积147.49万平方米，销售额29.47亿元，分别增长29.5%和33.1%。“万村千乡”市场工程建设加快，行政覆盖率达到70%，农村消费环境有效改善。

——农业生产平稳增长。克服了长时间低温冻害、连续降雨和强台风“黑格比”袭击等影响，农业生产稳步发展。实现农林牧渔业总产值371.32亿元，增长3.9%。罗非鱼、水东芥菜等特色农业发展迅速。新增国家重点农业龙头企业1家、省级农业龙头企业2家、省级名牌农产品6个。农业标准化和现代化示范区建设成效显著。农业综合开发取得新突破，高州市荣获全省农业综合开发立项竞争第一名。新增农民专业合作社101个，有社员1.16万人。

——社会创业活力不断增强。顺利实施《茂名市财政管理体制调整方案》，县域经济发展活力增强，6个县（市、区）财政一般预算收入平均增长19.6%，明显高于市级增幅。新设信用担保机构2家。新登记内资企业367户，私营企业1008户，外商投资企业77户。新增广东省著名商标11件、省级民营科技企业5家，民营科技企业总产值54.1亿元，增长21.7%。

——城市建设有效推进。完成了《茂名市城市总体规划（2008—2020）纲要》修编和茂港中心城区、站南片区等分区规划，成功举办了环水东湾新城规划国际招标，进一步明确了城市发展方向和空间。红旗路、站前路、茂名大道等道路改扩建工程开工建设。完成交通基础设施建设总投资9.9亿元，新建改建公路1516.4公里。市区新增公共绿地面积11.95万平方米，绿化覆盖率达41.3%。洛湛铁路茂名段加快建设。茂湛铁路、沿海铁路茂名段、博贺新港区铁路支线以及包茂高速茂名段前期工作抓紧推进。锡塘生活垃圾填埋场完成前期工作，完成水东港区2万吨级航道疏浚，3万吨级综合码头和1万吨级粮油综合码头建成投用。

——改革开放继续深化。茂名大厦等国有企业改革稳步推进，县级畜牧兽医体制、公安机关机构改革已经完成。农村信用社改革取得阶段性成果，成功取得央行17.05亿元专项改革票据。代建制试点工作有效推进。推行事业单位用人公开招聘。引进了174名高层次人才。财政体制改革扎实有效。对外开放迈出新步伐。全年外贸出口总值4.95亿美元，增长24.7%；实际利用外资5565万美元，增长11.1%；新批外资项目42宗，增长20％。

——节能减排和环境保护工作取得新成效。高州水库生态保护力度加大。环境污染得到有效控制，环境质量保持良好，饮用水源水质达标率保持100%，市区空气环境质量不断改善。全市化学需氧量和二氧化硫排放总量分别下降5.25%和7.64%，规模以上工业增加值能耗下降6.5%。

——人民生活有效改善。城市居民人均可支配收入和农民纯收入分别为12006元和5405元，增长7.2%和7%。居民消费价格总水平累计上涨5.6%，物价涨幅得到有效控制。新增城镇就业岗位5.26万个，城镇登记失业人员实现再就业2.3万人，年末城镇登记失业率3.17%，实现农村劳动力转移就业103万人，就业局势保持稳定。抗灾救灾取得重大胜利，因灾造成的5520户房屋“全倒户”全部动工重建。对口支援汶川县银杏乡重建工作进展顺利，全市共接收捐款6327.5万元，学校、医院等首期项目启动建设。社会保险覆盖面进一步扩大，社会保障体系不断完善。启动城镇居民基本医疗保险、生育保险工作，失地农民社会保障纳入城镇社保体系，妥善解决了早期离开国有集体企业人员的社保参保难问题。在我市居住的农民全部参加了新型农村合作医疗。16.8万城乡居民纳入低保范围，低保标准提高到城镇居民每月170～228元、农村居民每月100元。市区第二期廉租房交付使用，共筹集廉租房建设资金2783万元，累计为1972户城镇低收入家庭提供了住房保障。住房公积金的住房保障作用得到强化，全市共提取和放贷6.48亿元，受益职工近万人。农村饮水安全工程完成投资1.27亿元，解决了33.47万人的饮水安全问题。

——社会事业协调发展。城乡免费义务教育全面实施，农村义务教育学校危房改造全面完成，城乡办学条件进一步改善，教育扶贫助学力度加大。“普九”水平得到巩固提高，高中阶段毛入学率达62.3%，高考上省大专线以上人数连续7年排全省第二。茂名学院、市一中、茂名职业技术学院征地工作基本落实。职业技术教育综合实力跃升全省前列。高等教育协调发展。科技对经济发展的贡献率不断提高，全年列入省级科技项目56项；高州市通过科技部科技富民强县项目验收。特色文化加快发展，文化基础设施和文化产业建设得到加强，文艺创作、新闻出版等取得新成效。成功举行第九届体育节，体育工作扎实推进。城市社区卫生服务网络不断扩展，重大疾病预防和卫生应急工作得到加强。第一批30所乡镇薄弱卫生院改造工程顺利推进。人口和计划生育工作进一步加强，完成省下达的人口计生任务。国防动员、民兵预备役和拥军优抚安置工作不断进步。外事侨务、民族宗教、对台、殡改、统计、打私、气象、防震、经济研究、方志、档案、驻外联络等工作取得新成绩。老龄、妇女、儿童、助残等事业持续发展。

——民主法制建设取得新成效。坚持向人大及其常委会报告工作、向政协通报情况，加强与民主党派、工商联、无党派人士和人民团体的联系，自觉接受监督。全年办理人大代表建议125件，政协提案307件，办理质量不断提高。切实加强应急管理工作，应对突发事件能力不断提升。抓好信访工作，一批信访突出问题得到解决。普法工作深入开展，大调解新格局初步建立。顺利完成村（居）“两委”换届选举，城市社区管理不断加强。深入推进平安茂名建设和公安“三基”工程，被授予“全国科技强警示范城市”。社会秩序保持稳定，各类案件持续下降，没有发生大规模的群体性事件和群死群伤的恶性事故。交通运输市场秩序得到整治。食品药品安全、安全生产工作进一步加强。全面推进依法行政，规范行政审批，提高政府工作透明度。认真开展机关作风建设活动，建成市、县两级行政审批电子监察系统，行政监察、审计监督得到加强，廉政建设和反腐败斗争深入推进。政府驾驭市场经济、加强社会管理的能力不断增强。

各位代表！一年来取得的成绩来之不易。这是在上级党委、政府和市委的正确领导下，全市人民共克时艰、奋力拼搏的结果。在此，我代表市人民政府，向全市人民，向中央、省驻茂单位和驻茂解放军、武警部队官兵，向各民主党派、人民团体以及社会各界人士，表示衷心的感谢！向所有关心和支持茂名发展的香港、澳门特别行政区同胞、台湾同胞、海外侨胞和国际友人，表示衷心的感谢！

回顾一年来的工作，我们清醒地看到，我市经济社会发展仍然存在不少薄弱环节，加上受国际金融危机影响，我市经济发展面临着严峻的形势。主要是：经济增速有所放缓，财政收支压力明显加大；投资规模和引资规模仍然偏小；现代产业体系建设任重道远，工业经济效益下滑，部分企业生产经营困难加剧；现代农业发展缓慢，农业增效、农民增收的困难增多；县域经济依然薄弱，自我保障与发展的能力不强；基础设施建设滞后于发展需要；自主创新能力弱，节能减排压力较大；城市管理亟待加强；投资环境亟待改善，转变政府机关作风任务艰巨；社会事业发展水平偏低。对这些问题，必须认真采取措施加以解决。

2009年工作安排

今年是新中国成立60周年、茂名建市50周年，是推动我市争当东西两翼实践科学发展观排头兵的关键一年。由于国际金融危机和国内经济下行压力加大的影响，我市也同样处于新世纪以来经济发展最为困难的时期。面对危机与困难，我们绝不能掉以轻心；面对危机与困难，我们必须迎难而上；面对危机与困难，我们更需要危中求机，在危机中突破，在危机中跨越。综观形势，当前我市机遇大于挑战，希望大于困难。

——中央和省采取扩内需、保增长的政策措施，有利于我市改善投资偏小的现状，为我市加快基础设施建设、进一步改善民生提供重大机遇。

——积极的财政政策和适度宽松的货币政策，有利于解决困扰已久的融资难问题，为我市争取各方支持创造了前所未有的机会。

——石化产业调整振兴规划明确我市作为全国九大石化基地之一，有利于我市进一步推进主导产业精深发展，为巩固和提升我市作为广东石化产业龙头地位提供了难得的契机。

——《珠江三角洲地区改革发展规划纲要》的实施，有利于我市加强与该地区的要素流动，为我市构建现代产业体系、提高劳动人口素质提供了有效途径。

——近年来我市注重基层基础，前瞻做好规划，干部群众人心思进，在危机面前同舟共济，有利于进一步统一思想，为我市未来发展奠定了良好的基础，注入了强大的动力。

特别是今年工作的开局令人振奋。我市在一些涉及长远发展的项目上有重大突破。省政府认定了我市石化产业转移工业园，上级批复了茂名港总体规划，省发改委同意我市开展博贺新港区和进港铁路的前期工作，几大国有商业银行对我市的融资政策放宽。我们完全有理由相信，我市必将迎来加快发展、科学发展、和谐发展的春天！

今年政府工作的总体要求是：全面贯彻党的十七大、十七届三中全会、省委十届四次全会以及市委九届六次全会精神，深入贯彻落实科学发展观，按照省“三促进一保持”和“十个着力”的要求，把保持经济平稳较快发展作为今年政府工作的首要任务，把扩大内需作为保增长的根本途径，把调整产业结构、建设三次产业协调发展的现代产业体系作为保增长的主攻方向，把深化改革、扩大开放作为保增长的强大动力，把改善民生作为保增长的出发点和落脚点，把抓好工作落实作为保增长的关键，促进经济社会又好又快发展。

今年经济社会发展的主要预期目标是：生产总值增长9.5%，单位生产总值能耗下降3.2%，社会消费品零售总额增长14%，全社会固定资产投资增长20%，外贸进出口力争有所增长，居民消费价格总水平涨幅控制在4%以内，地方财政一般预算收入增长9%，城镇登记失业率控制在4%以内，城市居民人均可支配收入增长9%，农民人均纯收入增长7%。

为实现上述目标，今年要突出抓好工作落实。抓落实既是一种认识、一种责任，也体现着能力、水平。各级政府各部门及其工作人员，必须把抓落实上升到事关我市发展兴衰的高度，创新落实的方法，在“明责任、抓规范、定进度、讲合作、突重点、重细节”等方面下功夫，增强紧迫感，提高服务意识，明确自身责任，制定周密可行的工作方案，调动各方面的积极性，抓住重点，一抓到底，注重细节，集中力量攻坚克难，务求取得实实在在的业绩。

今年要着力抓好以下八个方面的工作：

一、千方百计增加投入，积极扩大内需

抓好重点工业项目建设。加快推进2000万吨炼油改扩建、60万吨PX、煤制氢等重大项目的建设，进一步巩固茂名石化公司全国最大炼化一体化企业的地位。尽快上马粤电2×100万千瓦博贺电厂、茂名热电厂7号机组、油页岩矿电联营以及信宜中坳风电项目。加快推进重力石化装备制造扩建项目。

抓好基础设施建设。密切配合，促使洛湛铁路茂名段尽快开通。加快茂湛铁路茂名段开工建设。配合做好博贺新港区铁路支线、沿海铁路、汕湛和包茂高速茂名段项目建设前期工作。加快博贺新港区深水港公用设施和进港公路建设，抓好茂名学院、市二技扩建以及茂名职业技术学院、市第二职业技术学校迁建工作。推进市一中新校区建设。

全年安排重点建设项目100项，总投资448亿元。

为加强项目工作，今年要采取以下措施：一要用足用好中央和省保增长的政策措施，抓紧谋划石化、电力、交通、水利、基础设施、能源资源储备等重点项目，争取更多项目纳入上级规划。二要积极争取中央、省属大型国有企业在我市布点新的重大项目，积极吸纳社会资金参与重点项目建设。三要强化重点项目建设的管理，统筹重点项目征地拆迁工作，落实责任，做好服务，提高项目开工率，确保项目建设顺利推进。

积极扩大消费需求。完善促进就业、工资正常增长、强农惠农等机制，促进城乡居民增加收入。积极培育消费热点，拓展消费空间。引导和促进汽车合理消费。扩大文化娱乐、体育健身等服务消费。做好“家电下乡”、“农机下乡”等工作，深入实施“万村千乡”市场工程，支持大中型流通企业向小城镇延伸经营网络，扩大农村地区消费。依法打击扰乱市场秩序的行为，维护消费者合法权益，营造安全放心的市场环境。加强市场价格监管，维护价格秩序和价格总水平基本稳定。

二、构建现代产业体系，提高产业发展水平

做强重化工业。抓住国务院出台石化产业调整振兴规划、我省大力实施《珠江三角洲地区改革发展规划纲要》的契机，进一步做大做强重化工业。重点要采取以下措施：一要积极做好服务工作，支持茂名石化公司发展壮大。二要密切与茂名石化公司的合作，抓紧编制石化产业规划，争取中石化的支持，吸引各方投资者，发展石化后续加工业。三要抓紧推进石化工业区和博贺新港区的建设，把两大工业区打造成沿海石化产业发展的重要基地。四要加大扶持力度，发展壮大装备制造业和电力工业。

大力承接产业转移。高起点、高标准建设珠海（茂名）产业转移工业园，创建全省示范性产业转移园区。支持各县（市、区）加快建设产业转移园区，改善园区基础设施，提高产业承接能力，促进主导产业和特色产业的发展。今年将安排产业转移工业园基础设施建设贷款贴息1亿元。

促进中小企业稳定发展。进一步清理和规范涉企收费，减轻企业负担。推进银企合作，加快建立信用担保机构，改善企业融资状况，推动企业上市融资。加强对企业的跟踪服务，及时应对异常情况。今年市级财政拟安排中小企业发展专项资金1000万元，帮助企业渡过难关，稳步发展。全力支持民营经济做大做强，实施“中小企业成长工程”，加大政策扶持力度，推进产学研合作，提高企业自主创新能力和市场竞争力，促进产业集聚和升级。鼓励和引导企业创立品牌，提高竞争力。建立健全现代企业制度。做好人才培养、引进和使用，为经济发展提供人才支撑。

大力发展现代服务业。改善服务业发展环境，加大财税金融支持力度，放宽市场准入。落实住房建设规划，出台扶持房地产业发展的政策措施，加强调控和引导，规范市场秩序，推行规模化的综合开发和配套建设，提高房地产开发水平。完善住房公积金制度，强化监管，落实住房消费优惠政策，扩大普通商品住房消费，加快保障性住房建设，促进房地产业稳定健康发展。发展商贸物流业，建设大型农、林、渔产品市场，优化城区商业网点布局，培育城区商业中心和特色商业街。重视城市旅游形象包装，建设旅游精品项目，重点打造生态休闲旅游、海滨旅游、温泉旅游、冼太文化等人文古迹旅游，提高旅游业发展水平，扩大“引客入茂”，实行国民旅游休闲计划。积极发展金融、会计、法律、咨询、物业、家政等现代服务业。

提高县域产业发展水平。落实鼓励县域经济发展的政策措施，调动县（市、区）的积极性，增强县域经济发展活力。实施“回归工程”，鼓励外出人员回乡创业。支持县（市、区）加强工业园区建设。鼓励县（市、区）接受市区辐射，大力发展石化产品后加工，形成更强的集群效应。发展农副产品深加工、海洋资源加工工业，壮大劳动密集型特色产业。推进各地专业市场、物流基地的规划与建设，形成完善的现代服务业体系。

三、加快农业基础设施建设，促进农村改革发展

抓好农业基础设施建设。推进高州水库灌区续建配套与节水改造、东南部沿海地区治旱调水等工程的建设，做好水库除险加固、中小型灌区改造、大中型水闸改造以及江(海)堤围加固达标工作。推进中低产田改造。

发展现代农业。稳定发展粮食生产，确保粮食安全。加快发展特色农业，抓好种养业主导农产品的生产。引进大型企业，发展壮大农产品流通加工业。扩大罗非鱼生产加工，建设全国最大的罗非鱼养殖加工出口一体化产业基地。推行农业标准化生产，提高农产品质量和竞争力。扶持农民专业合作社加快发展，培育壮大农业龙头企业。积极争取上级在粮食、生猪、农业综合开发、农业农村基础设施、农村社会事业等方面对我市的重点扶持。

推进农村改革。稳定和完善农村基本经营制度，稳妥推进农村土地承包经营权流转，落实农民对承包土地的使用权和受益权。加快农村土地确权工作和集体林权制度改革。推进农村金融改革，引导信贷资金和社会资金投向农村。

努力增加农民收入。全面落实中央和省的强农惠农政策，加大“三农”投入。抓好农村劳动力培训和转移就业工作，创新劳务输出工作机制，打造劳动力输出品牌。引导农民开展生态、休闲、观光等多种形式的农业经营活动。支持农民就地转移就业和创业，增加非农收入。引导有条件的返乡农民工创业发展、参加农业和农村基础设施建设。

今年市级财政用于农林水事务的支出将比去年预算增长20.2%。

四、深化改革开放，增强发展动力

推进重点领域和关键环节的改革。按照省的部署完成新一轮政府机构改革，开展第四轮行政审批制度改革，精简和规范行政审批，稳步推进事业单位分类改革、公务员分类管理及公务员聘任制改革。落实好中央新出台的财税政策，完善县、镇两级激励型财政体制，推进预算管理体制改革、国库管理制度改革和“金财工程”建设，深化公有资产管理体制改革。深化国有企业改革，稳步推进药品、医疗服务等价格和路桥通行费等收费改革。执行资源性产品价格改革政策，推进污水处理、垃圾处理、矿产资源有偿使用等方面的收费改革。

千方百计招商引资。突出招商重点，引导各方资本投向我市重化工业、基础设施、农产品加工、矿产资源加工和服务业领域。积极争取国内外大型知名企业到我市投资兴业。充分调动县、镇招商引资的积极性，努力掀起新一轮招商热潮。今年重点要在做好项目包装的基础上，在珠三角地区举行两场大型招商会。清理和规范涉及外商投资的行政收费和检查事项，加强对企业的跟踪服务，稳定现有企业并鼓励其增资扩产。

积极扩大对外贸易。落实国家鼓励出口的政策，优化出口商品、主体和市场结构，努力扩大规模，提高效益。支持和引导企业扩大传统优势产品出口，增加农产品出口，培育品牌，提高产品附加值。扶持重点外贸企业和民营企业扩大出口。支持企业开拓国际市场。适度扩大重要原材料、能源资源、关键设备等商品进口，引进先进适用技术。鼓励有条件的企业到境外投资兴业。

五、建设宜居城乡，改善人居环境

加快城市发展步伐。以建设宜居海滨城市为目标，抓好城市规划建设工作。抓紧完善新版城市总体规划，修编土地利用总体规划和主体功能区规划，做好“三个规划”的衔接；抓好各级城镇总体规划、控制性详细规划、专项规划的编制，以科学的规划引领城镇可持续发展。加快环水东湾片区规划建设。抓好茂名大道、茂港高地片区城市基础设施、大洲岛大桥及吹填配套等项目建设，以组团、滚动发展模式推进城市发展，拉大城市框架。分步骤改造河西旧城区，稳妥推进城中村改造，完善现有城区道路网络、公用设施。优化城市绿化建设，迎接国家园林城市复检。支持县城、中心镇和建制镇加快发展。创新城市管理模式，整合市、区、街道三级城市管理资源，统筹综合执法与部门执法，加强社区建设管理，努力提高城市品位和吸引力。

建设宜居新农村。加强农村基础设施建设，今年内完成老区镇通村公路硬底化。做好“告别泥砖房”工程收尾工作。推进农村饮水安全工程和村庄整治，开展农村垃圾集中收集处理试点，发展沼气、太阳能等清洁能源，改善农村人居环境。

加强节能减排和环境保护。严格实行节能减排问责制和“一票否决”制。推行清洁生产，发展循环经济，压缩淘汰落后产能。推广使用节能产品，推进建筑节能。加快建设县（市、区）污水处理厂，推进工业污水处理及排海工程、垃圾焚烧发电、锡塘生活垃圾填埋场等项目建设。严密监管工业污染源，开展环保专项行动，打击环境违法行为。认真整治鉴江、小东江沿岸和高州水库周边水污染源，加强饮用水源水质的监管。今年市级财政还将投入1000万元，用于高州水库水源保护工作。加强农村环境管理和生态保护。完善激励和约束机制，做好节约集约用地工作。

推进林业生态市建设。实施林业生态市建设规划，优化林业生态建设布局，加快造林绿化步伐。抓好大雾岭、鹿湖顶、林洲顶、河尾山等重点自然保护区建设，创建国家级自然保护区。加强水源林生态保护，完善和落实生态补偿机制。积极探索生态公益林的经营管理新模式。

六、抓好财税金融工作，增强经济发展支撑能力

做好财政增收工作。积极培育新的税收增长点，加强税收征管，促进税收稳定增长。完善非税收入征管，严格执行“收支两条线”。坚持依法治税，严肃处理涉税违法违纪行为。积极争取中央、省的扶持资金。

优化财政支出结构。按照保运转、保重点、保民生和集中财力办大事的原则，优化财政资源配置，压缩一般性行政支出。坚持勤俭办事，开展行政经费节约年活动，比照近3年平均基数，今年因公出国出境经费、公务用车经费、公务接待经费分别削减20%、15%和10%。坚持审账拨款、资金跟踪审计，推进财政支出绩效评价工作，提高财政资金使用效益。加强政府性债务管理，防范债务风险。

增强金融服务能力。加强社会信用体系建设，改善金融生态环境，引导金融机构加大对地方经济建设的信贷支持，促进地方金融业稳健发展。加强与商业银行的合作，逐步化解我市多年积累的银行呆坏账，提高我市融资能力。深入推进农村信用社改革，鼓励创办村镇银行和小额贷款公司。

七、着力解决民生问题，推进和谐社会建设

千方百计扩大就业。落实积极就业政策，保持就业形势稳定。培育新的就业增长点，鼓励优势劳动密集型企业发展，开发社区就业岗位和公益性岗位，全年新增城镇就业岗位5万个。把促进高校毕业生就业放在突出位置，落实扶持政策，引导高校毕业生到基层和企业就业。促进农村劳动力转移就业。改善对就业的公共服务，完善城乡就业援助制度，做好困难群体就业工作。推行劳动合同和集体合同制度，完善劳动关系三方协调机制，加强劳动保障监察执法和劳动争议处置。大力支持自主创业和自谋职业。

加快完善社会保障体系。完善城乡最低生活保障制度。推进社会保险扩面征缴，实现社保各险种市级统筹。全面落实新的养老金计发办法，扩大失业保险基金支出使用范围的试点，落实医疗保险政策，推动灵活就业人员、大学生及失地农民参加社会保险。做好优抚安置和救灾救济工作，健全社会救助体系。加强社保基金监管和退休人员社会化管理服务。为加快社会保障体系建设和促进就业，今年市级财政拟安排的社会保障和就业支出比去年预算增长16.7%。

大力发展社会事业。积极实施专利和名牌战略，加快科技服务体系和专业镇建设。抓好市区中小学和义务教育规范化建设，促进义务教育均衡发展。完善助学制度。加强师资队伍建设，扎实推动中小学教师工资福利待遇“两相当”工作，稳妥解决中小学代课教师问题。加快普及高中阶段教育，大力发展中等职业技术教育，提高高等教育发展水平，规范和促进民办教育。今年市级财政拟安排的教育支出比去年预算增长9.2%。鼓励发展文化事业和文化产业，重视特色文化建设，加强公共文化设施建设。推进医药卫生事业改革发展，完善公共卫生和医疗服务体系。提高城镇居民基本医疗和新型农村合作医疗保障水平。抓好乡镇薄弱卫生院改造，改善社区卫生服务。强化疾病防控和卫生监督执法。推进中医药强市建设。广泛开展群众性爱国卫生活动。今年市级财政拟安排的医疗卫生支出比去年预算增长8.4%。积极开展体育活动，加强公共体育设施建设，发展体育产业。落实人口和计生工作责任制，稳定低生育水平。抓好殡葬改革，推进绿色殡葬。深入实施妇女、儿童发展规划。做好残疾人工作，改善残疾人生活状况。关注老龄化的发展趋势，关心长者的身心健康。加强国防建设，做好“双拥”和民兵预备役工作。统筹做好审计、统计、外事侨务、民族宗教、对台、方志、档案、气象、地震、人防、驻外联络、慈善和红十字会等工作，推动社会全面进步。

维护社会稳定。推进“平安茂名”建设，健全社会治安防控体系，强化社会治安综合治理，对治安重点地区和突出问题进行整治，重视农村治安工作，依法严厉打击各类违法犯罪活动。做好信访工作，积极预防和妥善处置群体性事件，促进社会和谐稳定。开展整顿和规范市场秩序专项行动以及“质量和安全年”活动，强化产品质量和食品药品安全监管，加强安全生产和消防监管，坚决遏制重特大安全事故发生。健全应急机制，提高应对突发事件能力。

做好对口支援汶川县银杏乡恢复重建工作。加快交通、农田水利、教育卫生等援助项目建设，充分发动社会力量参与重建工作，着力改善灾区民生。

今年市级财政拟安排民生支出9.36亿元，比去年预算增长16.1%。

八、加强政府自身建设，进一步提高行政效能

加强政府民主法制建设。自觉接受人大及其常委会的监督，坚持定期向人大及其常委会报告重要工作。主动加强与政协的工作沟通和协商，接受其民主监督。积极听取各民主党派、人民团体和社会各界的意见和建议，支持工会、共青团和妇联等人民团体更好地发挥作用。提高人大议案、政协提案的办理质量和效率。巩固和扩大基层民主，深入推进村（居）民自治。加强基层队伍建设，今年市级财政拟安排村委会干部补贴1000万元，每人每月增加100元；继续补助全市103个镇，每镇10万元。

全面正确履行政府职能。紧紧围绕保增长、保民生、保稳定的大局，加强政府的经济和社会职能。强化经济运行监测和调节，妥善应对各种经济风险。切实改善对市场主体的服务，增强企业投资和社会消费的信心。强化政府的社会管理和公共服务职能，高度关注民生，切实解决涉及群众利益的难点、热点问题，促进基本公共服务均等化，维护社会安定团结。

提高政府效能。全面推进依法行政，严格按照法定权限和程序行使权力，规范行政行为。以机构改革为契机，创新政府运行机制和管理方式，理顺部门职责关系，加强部门协作，确保政令畅通，切实提高政府执行力。推进政务公开和电子政务建设，发挥市行政审批电子监察系统的作用，提高办事效率，改善投资环境。深入开展政府机关作风建设活动，抓好公务员队伍管理，大力建设服务型机关。加强廉政建设和反腐败工作。强化行政监察、审计等专门监督，实行行政过错责任追究和行政首长问责制度。各级政府机关工作人员要恪尽职守，艰苦奋斗，共克时艰。

各位代表！面对艰巨而繁重的工作任务，我们充满必胜的信心。让我们锐意进取，扎实工作，为完成今年的工作目标、推动茂名科学发展而努力奋斗！
